# A retrospective analysis of melioidosis in Cambodian children, 2009–2013

**DOI:** 10.1186/s12879-016-2034-9

**Published:** 2016-11-21

**Authors:** Paul Turner, Sabine Kloprogge, Thyl Miliya, Sona Soeng, Pisey Tan, Poda Sar, Pagnarith Yos, Catrin E. Moore, Vanaporn Wuthiekanun, Direk Limmathurotsakul, Claudia Turner, Nicholas P. J. Day, David A. B. Dance

**Affiliations:** 1 Cambodia Oxford Medical Research Unit, Angkor Hospital for Children, Siem Reap, Cambodia; 2 Epidemic Diseases Research Group Oxford, Centre for Tropical Medicine and Global Health, Nuffield Department of Medicine, University of Oxford, Oxford, UK; 3 Mahidol-Oxford Tropical Medicine Research Unit, Faculty of Tropical Medicine, Mahidol University, Bangkok, Thailand; 4 Lao-Oxford-Mahosot Hospital-Wellcome Trust Research Unit, Microbiology Laboratory, Mahosot Hospital, Vientiane, Lao PDR; 5 Centre for Tropical Medicine and Global Health, Nuffield Department of Medicine, University of Oxford, Oxford, UK

**Keywords:** Cambodia, Melioidosis, Paediatric

## Abstract

**Background:**

Melioidiosis, infection by *Burkholderia pseudomallei*, is an important but frequently under-recognised cause of morbidity and mortality in Southeast Asia and elsewhere in the tropics. Data on the epidemiology of paediatric melioidosis in Cambodia are extremely limited.

**Methods:**

Culture-positive melioidosis cases presenting to Angkor Hospital for Children, a non-governmental paediatric hospital located in Siem Reap, Northern Cambodia, between 1^st^ January 2009 and 31^st^ December 2013 were identified by searches of hospital and laboratory databases and logbooks.

**Results:**

One hundred seventy-three evaluable cases were identified, presenting from eight provinces. For Siem Reap province, the median commune level incidence was estimated to be 28-35 cases per 100,000 children <15 years per year. Most cases presented during the wet season, May to October. The median age at presentation was 5.7 years (range 8 days–15.9 years). Apart from undernutrition, co-morbidities were rare. Three quarters (131/173) of the children had localised infection, most commonly skin/soft tissue infection (60 cases) or suppurative parotitis (51 cases). There were 39 children with *B. pseudomallei* bacteraemia: 29 (74.4%) of these had clinical and/or radiological evidence of pneumonia. Overall mortality was 16.8% (29/173) with mortality in bacteraemic cases of 71.8% (28/39). At least seven children did not receive an antimicrobial with activity against *B. pseudomallei* prior to death.

**Conclusions:**

This retrospective study demonstrated a considerable burden of melioidosis in Cambodian children. Given the high mortality associated with bacteraemic infection, there is an urgent need for greater awareness amongst healthcare professionals in Cambodia and other countries where melioidosis is known or suspected to be endemic. Empiric treatment guidelines should ensure suspected cases are treated early with appropriate antimicrobials.

**Electronic supplementary material:**

The online version of this article (doi:10.1186/s12879-016-2034-9) contains supplementary material, which is available to authorized users.

## Background

Meliodiosis, infection by the Gram negative environmental bacterium *Burkholderia pseudomallei*, is an important but frequently under-recognised cause of morbidity and mortality in Southeast Asia and elsewhere in the tropics [1, 2]. It was recently estimated that there might be as many as 165,000 cases of meloidosis worldwide in 2015, an annual incidence rate of 5 per 100,000 people at risk [3]. Infection occurs following per-cutaneous inoculation, inhalation, or ingestion of the organism. The spectrum of infections is broad, ranging from minor skin or soft tissue infection to pneumonia and bacteraemia with a high mortality. Parotitis is a common presentation in children in Southeast Asia [4–6]. This may be the result of ingestion or aspiration of *B. pseudomallei*-contaminated water sources [7]. Standard treatment of melioidosis consists of an acute phase consisting of intravenous ceftazidime or a carbapenem for at least 10 days followed by an eradication phase consisting of oral co-trimoxazole or co-amoxiclav for at least 12 weeks [8].

To date, there are few data on the epidemiology of melioidosis in Cambodia, a low-income country with a 2008 population of 13.4 million (including 4.5 million children aged less than 15 years) [9]. The country has extremely limited microbiology laboratory capacity. Vlieghe and colleagues described 58 adult cases presenting to a single hospital in the capital, Phnom Penh, between July 2007 and January 2010 [10]. Angkor Hospital for Children (AHC), a non-governmental hospital located in Siem Reap province, identified a total of 39 paediatric melioidosis cases over an overlapping time period (October 2005 to December 2008), following the opening of a diagnostic microbiology laboratory within the hospital [11]. Although these figures are lower than the numbers of cases seen annually in neighbouring Thailand [12], more recently, *B. pseudomallei* has been reported to account for 74% of suppurative parotitis [6], and 7.9% of culture-positive bacteraemia [13] seen in AHC, further evidence that melioidosis is likely to have been grossly underdiagnosed in Cambodia. Recent modelling has suggested that the true number of cases of melioidosis nationally may be around 2000 per year [3]. Assuming that between 5 and 15% of melioidosis occurs in children [4], 100–300 paediatric cases per year would be expected across the whole country.

The aim of this retrospective study was to describe the epidemiology, clinical presentations, treatment and outcomes of melioidosis in Cambodian children presenting to AHC over a 5-year period.

## Methods

### Study site

Cambodia has a tropical climate with monsoon rains occuring between May and October each year. Siem Reap province is located in the northwest of the country and comprises 12 districts and 100 communes. The province had a total population of 896,443, including 322,857 children aged <15y, according to the 2008 national census [9]. Angkor Hospital for Children is one of two paediatric hospital located in Siem Reap province. This non-governmental hospital comprises a main site in Siem Reap city and, since 2010, a smaller satellite clinic (SC) located in Sot Nikom district referral hospital, 35 km from the main hospital. AHC provides free secondary and tertiary level care to children aged <16 years with no geographic restriction. There are approximately 180,000 patient presentations and 7000 admissions per year; around 2/3 of children admitted reside in Siem Reap province. A microbiology laboratory is located at the main hospital and specimens are transported from the satellite clinic for processing on a daily basis. Microbiology specimens are routinely processed to identify *B. pseudomallei* infection as previously described [11]. Briefly, 1–4 ml blood specimens are cultured for up to 7 days in 20 ml tryptone soya broth (Oxoid, UK) plus 0.05% sodium polyanethol sulphonate (Sigma-Aldrich, UK). Bottles are inspected daily with sub-culture if the broth is cloudy and additional blind sub-cultures at 24 h and 7 days. Suspected *B. pseudomallei* colonies are confirmed by Gram stain, a *B. pseudomallei* latex agglutination test [14] and/or API 20NE (bioMerieux, France), plus resistance to colistin and gentamicin discs (Oxoid). Non-blood specimens are cultured onto a range of media, including Ashdown’s agar, and *B. pseudomallei* identified as described for blood cultures. AHC clinical guidelines cover identification and treatment of suspected and culture-confirmed melioidosis. Specifically, a blood culture is recommended in all cases of suspected melioidosis plus a throat swab in those with suspected pneumonia or sepsis and a urine culture in those with suspected sepsis. Abdominal ultrasound scanning is recommended if visceral involvement is suspected. Ceftazidime and/or imipenem were available for empiric treatment throughout the study period.

### Case definitions

A melioidosis case was defined as a patient in whom *B. pseudomallei* had been isolated from at least one clinical specimen collected between 1^st^ January 2009 and 31^st^ December 2013. Cases were identified by manual searches of laboratory logbooks and electronic databases. Results of these searches were cross-checked against the hospital electronic patient information system to identify and correct discrepancies. The English language medical records of cases were retrieved and clinical, laboratory, and radiology data extracted onto a standardised case report form (CRF).

Cases were defined as localised or disseminated as described elsewhere [11]. Briefly, localised infection was defined as a single anatomic focus of infection whereas disseminated infection was defined as localised infection in two or more discrete anatomic areas and/or *B. pseudomallei* bacteraemia. Suppurative parotitis was defined as infection of the parotid confirmed by ultrasound or surgical findings: cases clinically diagnosed as “parotitis” without such confirmation were categorised as skin/soft tissue infection. Secondary foci of infection were assigned by review of clinical, lab, and radiological data. Lymph node abscesses were considered secondary foci if there was a culture postive skin/soft tissue infection (SSTI) in an anatomically relevant location; SSTI were considered secondary foci in pneumonia cases; and otitis/mastoiditis was considered secondary to supurative parotitis. Cases in whom symptoms had been present for >2 months at presentation were categorised as chronic melioidosis [15].

### Data management

Completed CRFs were single-entered into a password protected database (Access 2013, Microsoft, USA). The data were checked for errors by systematic plotting of numerical datapoints and review of all outlying values plus a review of a subset of CRFs. Data were analysed using R version 3.20 [16]. Maps were drawn with QGIS version 2.8 [17], using Open Development Cambodia basemaps containing commune level population data from the 2008 Cambodian National Census [9, 18]. Nutritional status was assessed in children aged 0–10 years by calculating weight-for-age z-scores using WHO AnthroPlus version 1.0.4 [19]. Absence of height data precluded assessment of nutritional status in older children. Non-normally distributed continuous variables were described by the median, inter-quartile range (IQR), and overall range. Proportions were compared by the Chi-squared or Fisher’s exact test, as appropriate. Two-tailed *p*-values of <0.05 were used to indicate statistical significance in all comparisions.

## Results

**Fig. 1:**
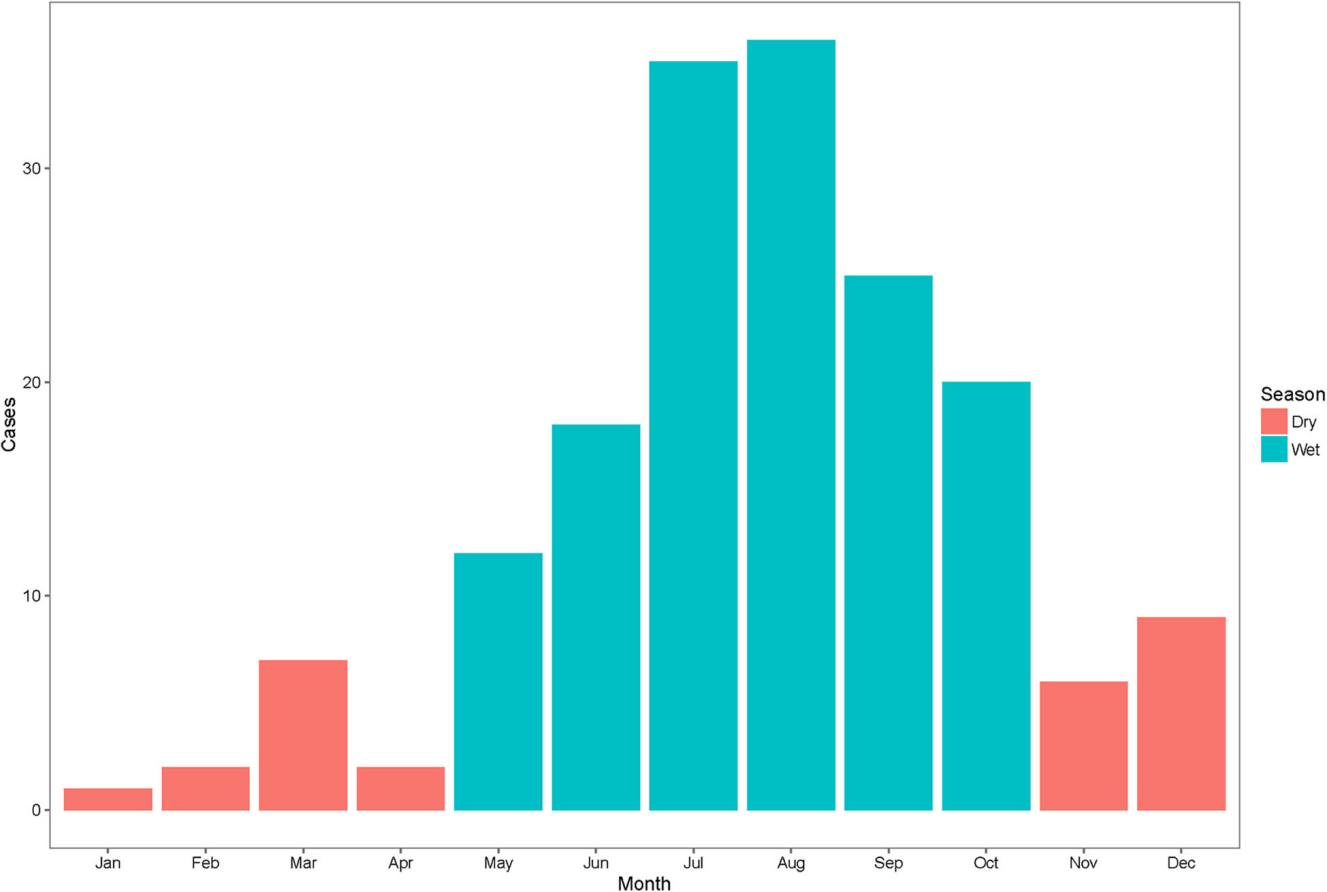
Seasonality of melioidosis case presentation at Angkor Hospital for Children, 2009–2013

Between 1^st^ January 2009 and 31^st^ December 2013, *B. pseudomallei* was identified in specimens from 176 patients. Medical records were available for review in 173 cases and these were included in the subsequent analyses (Additional file 1: Figure S1). Nineteen cases were identified in 2009, with between 35 and 44 cases in subsequent years (Additional file 1: Figure S2). Cases presented from eight provinces, with almost three quarters (72.8%; 126) of cases residing in Siem Reap province (Additional file 1: Figure S3). The distribution of cases within Siem Reap province (Additional file 1: Figure S4) was similar to the overall distribution of hospital admissions (Additional file 1: Figure S5) over the same time period. Over eighty percent (146/173) of melioidosis cases presented during the wet season, May to October (Fig. 1).

The median age at presentation was 5.7 years (IQR 3.1–9.5; range 0.02–15.9; Additional file 1: Figure S6). There were three neonatal cases and the youngest patient was 8 days old at first presentation. Seventy-four (42.8%) children were female. Only five children had significant co-morbidities documented: two with thalassaemia and one each with chronic renal failure, probable acute lymphoblastic leukaemia (ALL), and systemic lupus erythematosus (SLE). Additionally, one child may have had an underlying immunodeficiency given two previous episodes of *Chromobacterium violaceum* sepsis [20]. None of the children were known to be diabetic at presentation, or were subsequently diagnosed as such. A weight measurement was available in 131/134 children aged 0–10 years and 48.1% (63/131) of these had weight-for-age z-scores of < −2, indicating that they were moderate to severely underweight.

One hundred and fifty children (86.7%) presented directly to AHC from home, with 20 presenting following attendance at another healthcare facility (data missing in three cases). The median duration of symptoms at presentation was 7 days (IQR 5–14; range 1 day–7 months). Six children were categorised as having chronic melioidosis at presentation. Almost a quarter (42/173; 24.3%) of children had evidence of disseminated infection: most commonly bacteraemia with clinical or radiological evidence of pneumonia (Table 1). A head and/or neck lesion was identified in 112/173 (64.7%) children. *B. pseudomallei* was isolated from 197 specimens: 146 pus/swab cultures, 43 blood cultures, 5 sputum cultures, and 3 throat swab cultures (2 from children with pneumonia and 1 from a child with parotitis). The organism was isolated from a median of one (range 1–6) specimen per patient. Only 57 (32.9%) children had a blood culture taken at presentation; this was more likely in medical admissions (43/57, 75.4%) compared with surgical admissions (14/116, 12.1%), *p* < 0.0001. None of the children admitted to hospital had an abdominal ultrasound scan result recorded in the clinical notes or hospital radiology database.

**Table 1:** Foci of infection in 173 melioidosis cases presenting to Angkor Hospital for Children, 2009–2013

	Number	Percent
Principal focus of infection		
* Bacteraemic*	39	22.5
Pneumonia	29	16.8
No focus found	6	3.4
Parotitis	2	1.2
Skin/soft tissue infection	2	1.2
* Non-bacteraemic/Blood culture not done*	134	77.5
Skin/soft tissue infection	60	34.6
Parotitis	51	29.4
Lymph node abscess^a^	15	8.6
Pneumonia	4	2.3
Vaginal discharge	2	1.2
Otitis/mastoiditis	1	0.6
Periorbital cellulitis	1	0.6
Secondary foci of infection (in addition to a primary focus)		
Skin/soft tissue infection	4	
Otitis/mastoiditis	2	
Lymph node abscess	5	

^a^14 head/neck and 1 lower limb

Twenty-nine (16.8%) children died and 23 (79.3%) of these died within a week of presentation (the median time to death was 1 day from presentation). Three of the deaths could not be clearly attributed to melioidosis: one in-hospital death may have been due to a ventilator-associated pneumonia, one early post-discharge death in the child with probable underlying ALL, and one late (>6 months) post-discharge death in one of the neonatal bacteraemia/pneumonia cases. Bacteraemia was significantly associated with death: 28/39 (71.8%) of bacteraemic children died compared with 1/134 (0.7%) of non-bacteraemic children (*p* < 0.0001).

Ninety-five (54.9%) children were admitted to hospital and the 78 remaining children were managed entirely in the out-patient department. In those admitted, the median duration of admission was 9 days (IQR 2–13; range 0–70). Twenty-six children died during hospitalisation and one was discharged against medical advice. In those hospitalised, 25/91 (27.5%) were treated with inotropes and/or steroids for management of haemodynamic instability and 25/94 (26.6%) required mechanical ventilation. Fifty-one (53.7%) hospitalised children had a surgical procedure, mostly incision and drainage of an abscess. Antimicrobial treatment data were available on 91/95 admitted children. On admission, empiric treatment was variable and most commonly included ceftriaxone (35 children) and/or cloxacillin (32 children). Nineteen children were empirically started on ceftazidime. Overall, 81 of the admitted children received ceftazidime and/or imipenem for a median of 9 days (IQR 5–13; range 0–52). The median time from presentation to starting either ceftazidime or imipenem was 3 days (IQR 1–3; range 0–52). Seven of the children who died in hospital did not receive ceftazidime or imipenem, with antimicrobial treatment details missing in another four: six died within 24 h of presentation and melioidosis was not suspected in the seventh case until a positive pus culture was obtained 13 days after admission. All 68 children discharged from in-patient treatment received follow-on oral eradication treatment. Most children received a single oral antibiotic: 41 received co-amoxiclav (median duration 65 days (IQR 32–110; range 4–202)), 11 received co-trimoxazole (median duration 32 days (IQR 23–87; range 11–105)), and one received doxycycline (for 94 days). The remaining children received various combinations of co-amoxiclav, co-trimoxazole, and doxycycline. Four children were readmitted following discharge: none had a microbiologically-proven melioidosis relapse but one child was empirically re-treated with ceftazidime for 11 days.

Of the 78 children treated entirely as out-patients, all but one had localised melioidosis. A minor surgical procedure was performed in 63 (80.8%) children. Empiric antimicrobial treatment was highly variable but commonly included cloxacillin (49 children). Eighteen children were treated empirically with co-amoxiclav and six with co-trimoxazole. Overall, 67 (85.9%) of these children received an oral antibiotic with activity against *B. pseudomallei*. Co-amoxiclav monotherapy was prescribed for 33 children (median 82 days (IQR 28–109; range 6–137)) and co-trimoxazole monotherapy was prescribed for 19 children (median duration 21 days (IQR 15–79; range 8–176)). The median starting dose co-amoxiclav was 250 mg (IQR 250–500; range 170–1000), equating to 20 mg/kg/dose (IQR 15–24; range 12–30). For co-trimoxazole, the median starting dose (trimethoprim + sulphamethoxazole combined) was 360 mg (IQR 240–480; range 240–720), equating to a sulphamethoxazole dose of 20 mg/kg/dose (IQR 16–21; range 15–25). The other 15 children were prescribed combinations of co-amoxiclav, co-trimoxazole, and doxycycline.

Excluding the 26 children who died in hospital, the median duration of follow-up from presentation to final hospital discharge or out-patient visit was 39 days (IQR 10–91; range 0–224). Duration of follow-up was 3 months or more in 50 children but successful completion of at least 12 weeks of treatment and symptomatic recovery could be documented definitively in only 20 cases. An additional 32 children were seen in the hospital out-patient department at least 4 weeks following their final melioidosis-related visit (median time from final melioidosis-related visit to most recent out-patient visit: 710 days (range 56–1777). Of these, 12 were treated with a single agent oral-only regimen (seven co-amoxiclav and five co-trimoxazole) and eight treatment courses were of <12 weeks duration.

**Fig. 2:**
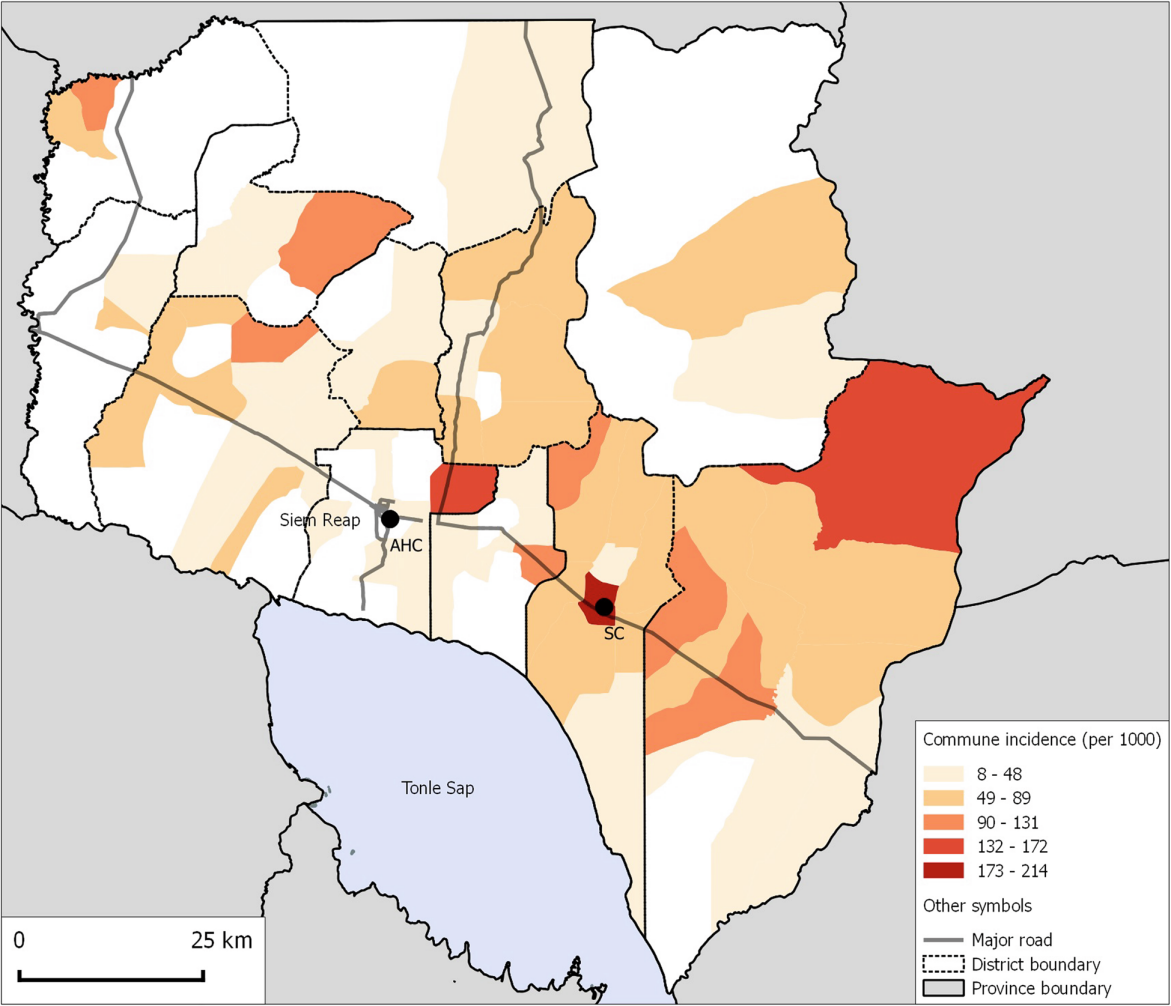
Commune-level incidence of melioidosis per 100,000 children aged <15 years in Siem Reap province over the 5-year study period (2009–2013). AHC: Angkor Hospital for Children; SC: AHC Satellite Clinic. Study data overlaid on the Open Development Cambodia basemap [18]

Disease incidence was estimated for the 61 communes in Siem Reap province with at least one melioidosis case over the study period, including the 125 cases aged <15 years and using commune <15 year-old population data from the 2008 national census as the denominator (Fig. 2). These 61 communes comprised 223,575 children aged <15 years (69.2% of all children <15y in Siem Reap province). Over the 5-year study period, the median commune-level incidence was 53 per 100,000 children (IQR 31–74; range 8–214). The median annual commune-level incidence ranged from 28 cases per 100,000 children (IQR 23–41; range 8–103) in 2011 to 35 cases per 100,000 children (IQR 27–55; range 15–129) in 2010 (Additional file 1: Figure S7). Overall incidence was re-calculated assuming that the 39 non-included communes were truly melioidosis-free. For the 5-year study period, this adjusted median level commune incidence would be 30 per 100,000 children (IQR 0–65; range 0–214).

## Discussion

This study represents the most comprehensive description of paediatric melioidosis in Cambodia to date. The data presented confirms the initial findings regarding paediatric melioidosis in Cambodia [6, 11, 13]. The majority of cases occurred in the monsoon season, were localised infections, and most children did not have underlying co-morbidities. Along with the adult data from Phnom Penh [10], the study highlights the wide geographic distribution of cases within the country. Unfortunately, despite greater awareness of the disease at AHC, the mortality in bacteraemic cases has not fallen significantly since the first cases were described: it was 77.8% (7/9 cases) between 2005–2008 and 71.8% (28/39) in the current study (2009–2013) [11]. Community-based awareness programmes, focussed on understanding disease risk factors and reducing time to presentation at hospital, may improve mortality. Consistent availability of ceftazidime for empiric treatment of suspected melioidosis is also critical: currently this may be sub-optimal in Cambodia as a result of cost. For AHC, the cost of a 1 g vial of ceftazidime is US$4.20, compared with US$1.05 for a 1 g vial of ceftriaxone. To put these costs in context, the GDP (gross domestic product) per capita for Cambodia was US$735 in 2009, rising to US$1020 in 2013 [21].

In Siem Reap province, the median annual incidence was estimated to be between 28 and 35 cases per 100,000 children <15 years per year. This is lower than the incidence in children in Ubon Ratchathani province, Northern Thailand: 48 cases per 100,000 children <15 years per year [12]. However, the Siem Reap figure is likely to be an underestimate for several reasons. Firstly, AHC is one of two paediatric referral hospitals in the province, meaning an unknown number of cases are likely to have been admitted and treated at the other facility. Secondly, microbiology specimens were sent for culture at the discretion of the attending clinician: this may have resulted in an underestimate in the total number of cases and also the proportion that were confirmed to be bacteraemic. Thirdly, given the difficulties of transportation in rural Cambodia, seriously unwell children may have been missed because of death prior to reaching hospital. Finally, a single census population estimate was used for the entire study period. Nonetheless, the fact that 35–44 cases of culture-positive melioidosis are being diagnosed at this single, relatively small paediatric hospital suggests that the recent estimate of 2000 cases per year in all age groups across the whole country may be conservative assuming that the incidence is similar in other parts of the country [3]. One other interesting finding is that there appear to be some relative ‘hot-spots’ of higher melioidosis incidence within the catchment area of AHC. It is notable that one of these coincided with the location of the AHC satellite clinic, suggesting the possibility of some referral and ascertainment bias, although this could not explain the other ‘hot-spots’ identified. A better understanding of the factors that contribute to such variations in incidence, for example whether this is related to specific local environmental or climatic conditions, differences in human behaviour or susceptibility, or the presence of unusually virulent bacterial strains, would be invaluable to our understanding of the epidemiology of melioidosis and the ecology of B. *pseudomallei*, and might help to predict other places where melioidosis hot spots could occur.

The study highlights the difficulties of empiric treatment and follow-up in low-resource settings such as Cambodia. Only 19/95 (20.0%) of the hospitalised children were treated empirically with ceftazidime and at least seven children did not receive an antibiotic active against *B. pseudomallei* prior to death. The duration of intensive treatment was also shorter than the recommended minimum of 10 days in many cases [8]. Oral antimicrobial dosing was variable: the median initial co-amoxiclav dose of 20 mg/kg was adequate [8], but the median initial co-trimoxazole dose of 20 mg/kg (sulphamethoxazole component) was significantly lower than the 30 mg/kg recommended in the Royal Darwin Hospital paediatric melioidosis guideline [15]. Successful completion of eradication treatment was only documented in 20/146 (13.7%) children surviving to hospital discharge. Similarly poor adherence to follow-up has been described in Indian children [22]. Creative models for follow-up for such populations such as home- or village-based review and monitoring of eradication treatment may be required in rural areas. There is a need for dedicated trials to determine the efficacy of oral-only and/or shorter treatment protocols for mild infections.

Similar paediatric case reviews have been completed, for example in Australia (45 cases between 1989 and 2013 [15] and 8 cases between 1996 and 2006 [23]), India (11 cases from 2007 to 2014 [22]) Malaysia (16 cases between 1976 and 2005 [24] and 13 cases between 2000 and 2003 [25]), Singapore (17 children between 2002 and 2014) [26] and Thailand (55 cases between 1979 and 1993 [27], 37 localised cases between 1994 and 2006 [28], and 46 cases between 1990 and 1991 [29]). Broadly the findings are similar in all studies. Around a third of cases presented with suppurative parotitis, which is in line with data from Thailand [5]. However, the mortality in Northern Australian children was significantly lower than in Cambodian children: 7% (3/45) compared to 17% (29/173). A small number of Australian children presented with neurologic manifestations (brainstem encephalitis). There was no evidence of neurologic involvement in any of the Cambodian children but there has been a single case of culture-confirmed *B. pseudomallei* meningitis at AHC since 2013.

The major limitation of the study was its retrospective nature, resulting in limited ability to explore risk factors for infection or poor outcome. Potentially important co-morbidities and environmental exposures may not have been documented reliably. Absence of abdominal ultrasound scan results meant that the frequency of intra-abdominal organ involvement could not be established. It was also not possible to explore the outcomes of children treated exclusively with oral antibiotic regimens. This would have been desirable as, to date, there are only scant data to support such regimens [4, 30]. Finally, data were obtained from a single hospital and so extrapolation of findings to the wider population of Cambodia must be done cautiously.

## Conclusions

This retrospective study demonstrates a considerable burden of melioidosis in Cambodian children. Given the high mortality associated with bacteraemic infection, there is an urgent need for greater awareness amongst healthcare professionals in Cambodia and other countries where melioidosis is known or suspected to be endemic. Empiric treatment guidelines should ensure suspected cases are treated early with appropriate antimicrobials. Attention should be paid to optimising adherence to long eradication treatment regimens following discharge from hospital.

## Additional file

Additional file 1: Figure S1. Study flow chart. **Figure S2.** Number of culture-confirmed melioidosis cases presenting to Angkor Hospital for Children, 2009–2013. **Figure S3.** Commune-level geographic distribution of culture-confirmed melioidosis cases from all provinces presenting to Angkor Hospital for Children, 2009–2013. **Figure S4.** Siem Reap province commune-level geographic distribution of culture-confirmed melioidosis cases admitted to Angkor Hospital for Children, 2009–2013. **Figure S5.** Siem Reap province commune-level geographic distribution of all patients admitted to Angkor Hospital for Children, 2009–2013. **Figure S6.** Age distribution of 173 melioidosis cases presenting to Angkor Hospital for Children, 2009–2013. **Figure S7.** Annual Siem Reap commune level melioidosis incidence estimates in children aged <15 years (PDF 737 kb)

## Abbreviations

AHC: Angkor Hospital for Children
ALL: Acute lymphoblastic leukaemia
CRF: Case report form
GDP: Gross domestic product
IQR: Inter-quartile range
SC: Satellite Clinic
SLE: Systemic lupus erythematosus
SSTI: Skin or soft tissue infection
WHO: World Health Organization
