# Supplementary material for: A retrospective analysis of melioidosis in Cambodian children, 2009–2013
Source: BMC Infect Dis. 2016 Nov 21;16:688. doi: 10.1186/s12879-016-2034-9 (PMC5117556; doi:10.1186/s12879-016-2034-9)

Supporting Information

A Retrospective Analysis of Melioidosis in Cambodian Children, 2009 – 2013

Paul Turner, Sabine Kloprogge, Thyl Miliya, Sona Soeng, Pisey Tan, Poda Sar, Pagnarith Yos, Catrin E Moore, Vanaporn Wuthiekanun, Direk Limmathurotsakul, Claudia Turner, Nicholas PJ Day, David AB Dance

Figure S1. Study flow chart

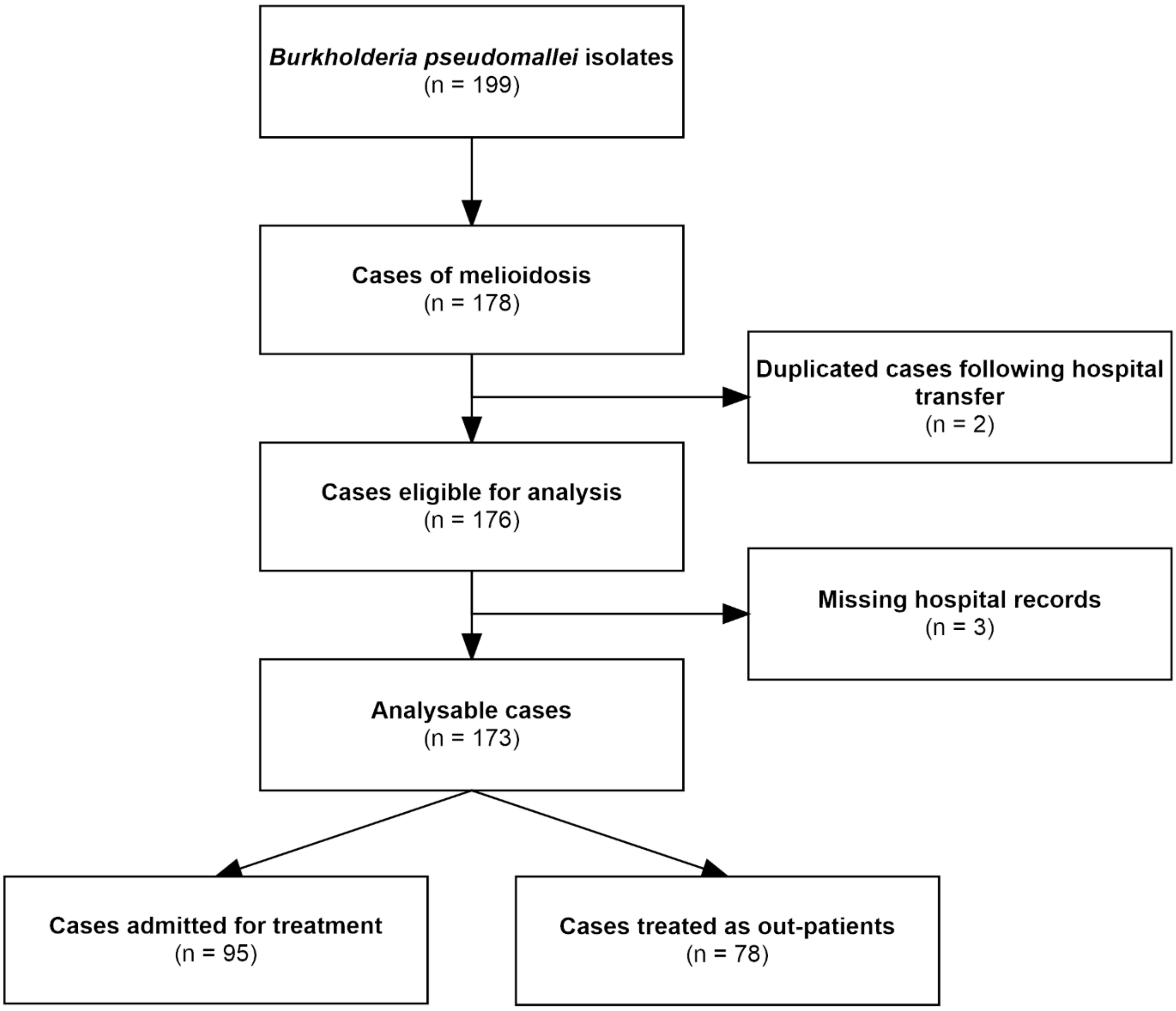

Figure S2. Number of culture-confirmed melioidosis cases presenting to Angkor Hospital for Children, 2009 - 2013

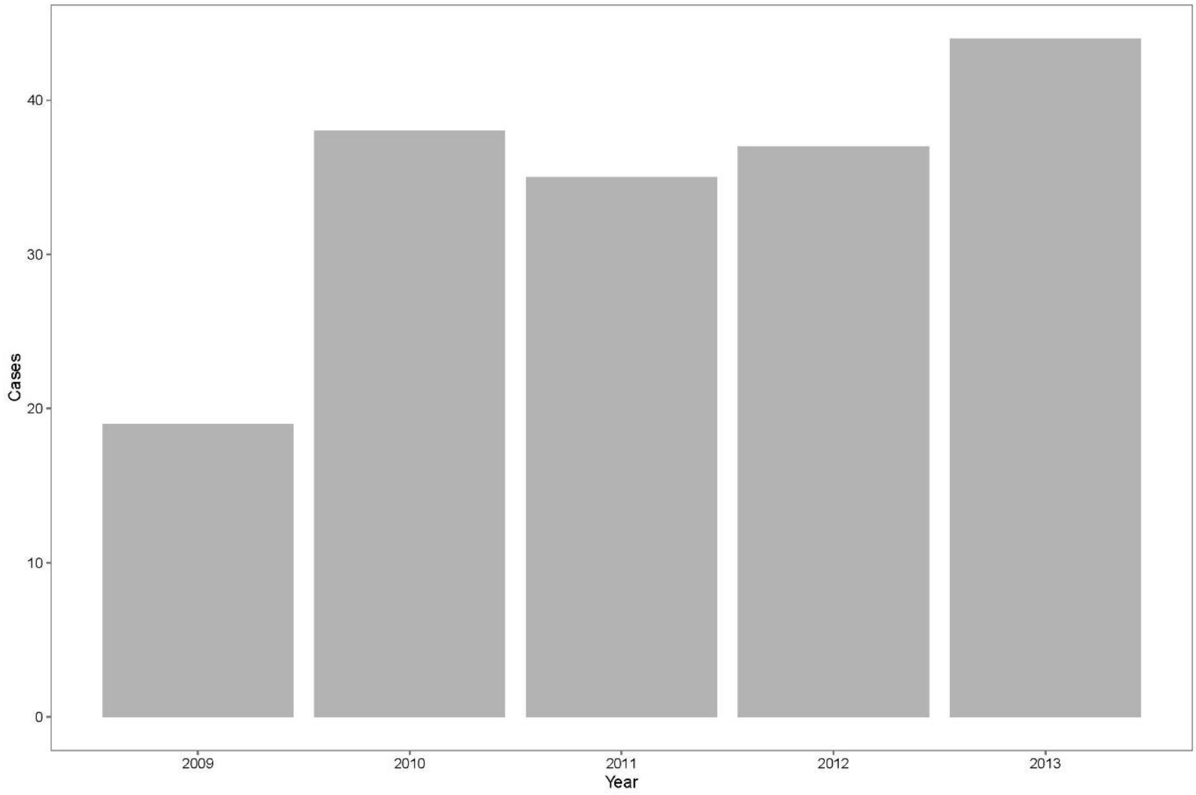

Figure S3. Commune-level geographic distribution of culture-confirmed melioidosis cases from all provinces presenting to Angkor Hospital for Children, 2009 – 2013.

Patients were mapped to the commune-level using data from the Cambodia National Census, 2008. Cambodia is shaded light grey with surrounding countries shaded dark grey.

Study data overlaid on the Open Development Cambodia basemap

[http://www.opendevelopmentcambodia.net/maps/downloads/. Accessed 1st July 2016.]

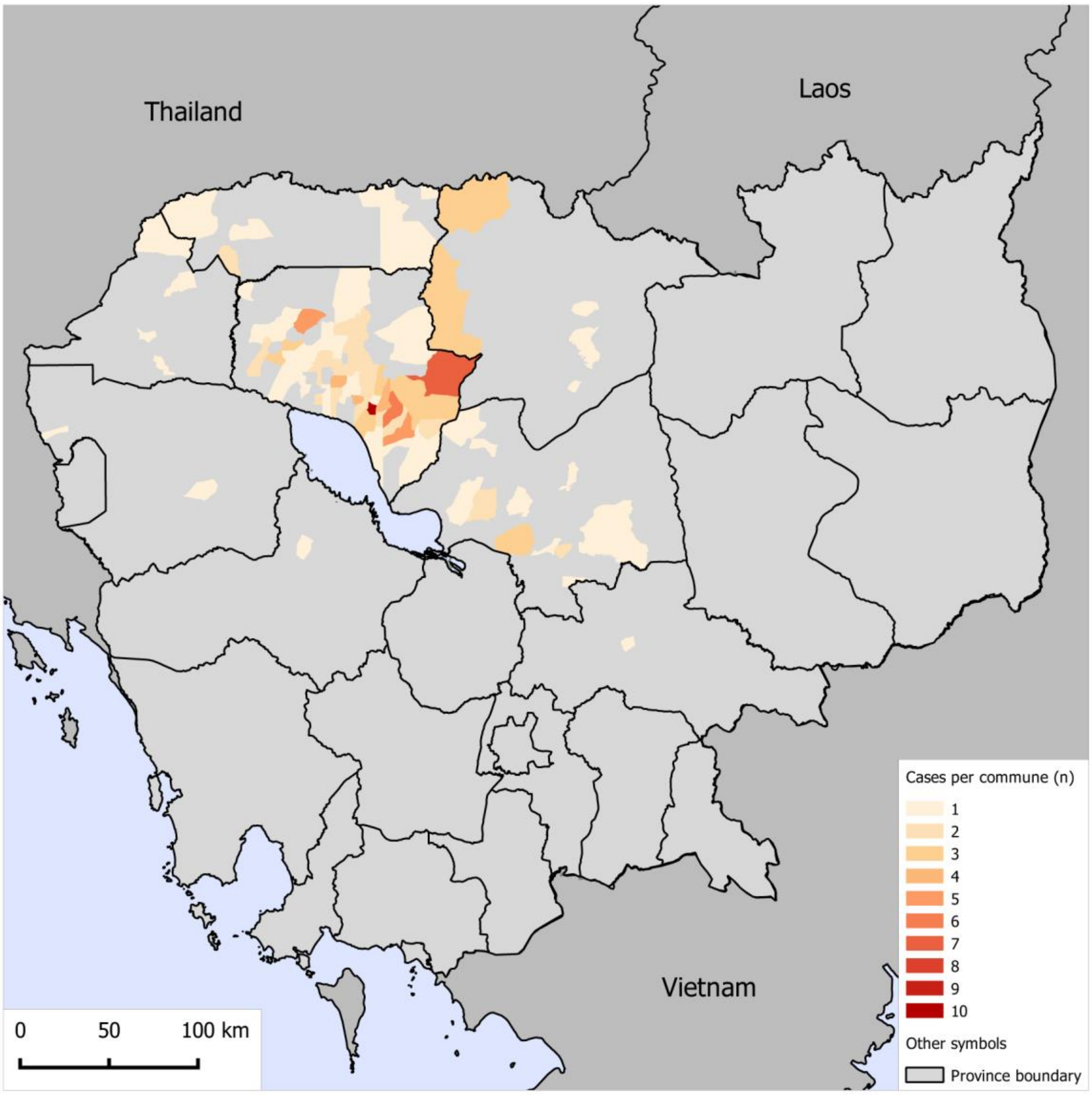

Figure S4. Siem Reap province commune-level geographic distribution of culture-confirmed melioidosis cases admitted to Angkor Hospital for Children, 2009 – 2013

Patients were mapped to the commune-level using data from the Cambodia National Census, 2008. AHC: Angkor Hospital for Children; SC: AHC Satellite Clinic

Study data overlaid on the Open Development Cambodia basemap [http://www.opendevelopmentcambodia.net/maps/downloads/. Accessed 1st July 2016.]

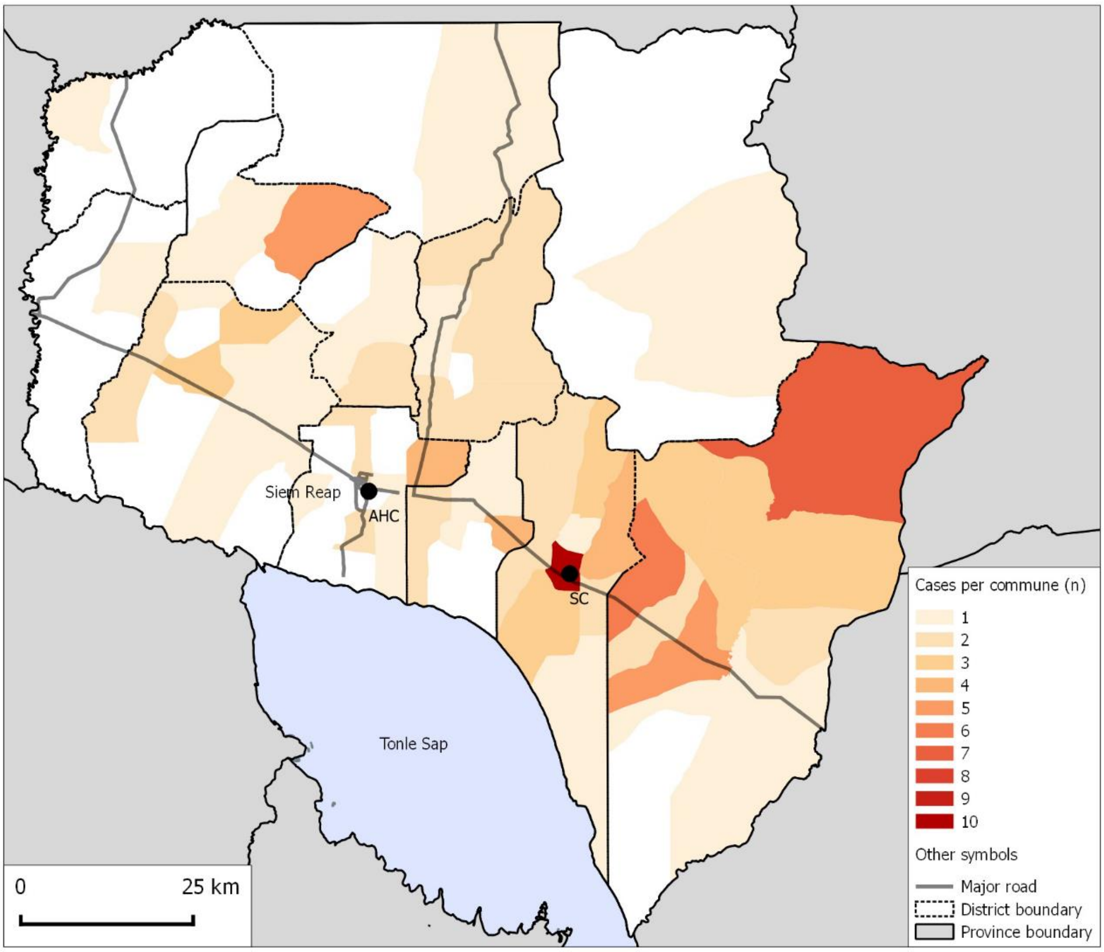

Figure S5. Siem Reap province commune-level geographic distribution of all patients admitted to Angkor Hospital for Children, 2009 – 2013

Patients were mapped to the commune-level using data from the Cambodia National Census, 2008.AHC: Angkor Hospital for Children; SC: AHC Satellite Clinic

Study data overlaid on the Open Development Cambodia basemap [http://www.opendevelopmentcambodia.net/maps/downloads/. Accessed 1st July 2016.]

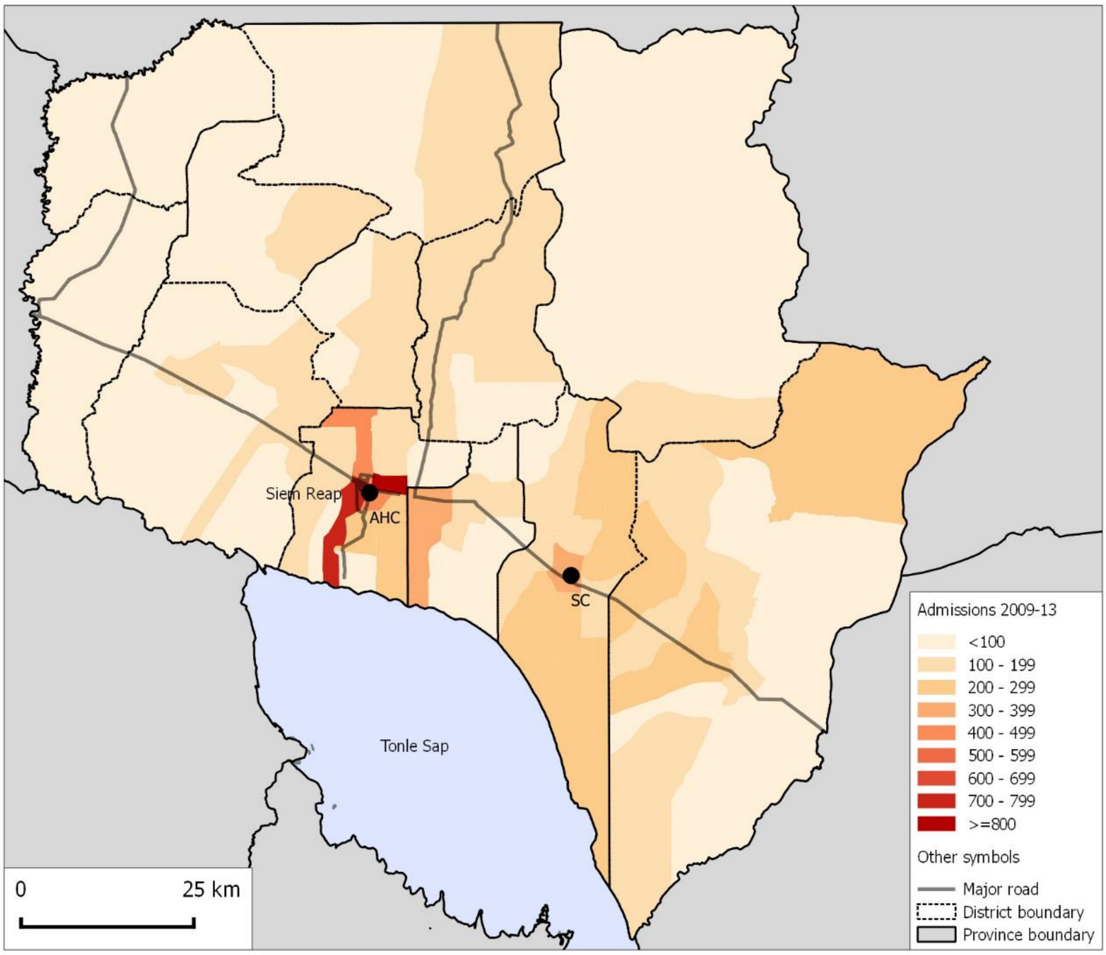

Figure S6. Age distribution of 173 melioidosis cases presenting to Angkor Hospital for Children, 2009 – 2013

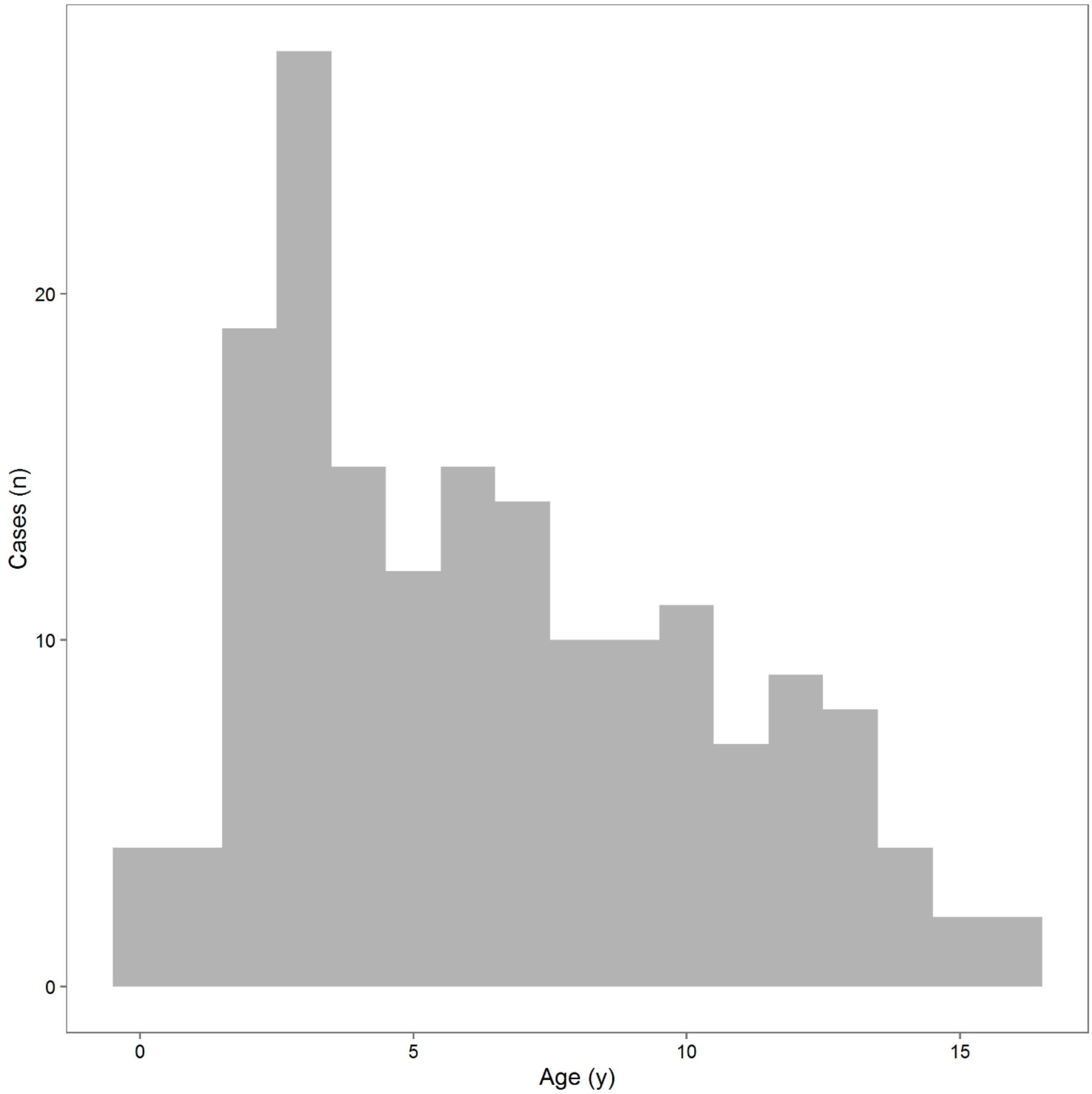

Figure S7. Annual Siem Reap commune level melioidosis incidence estimates in children aged <15 years

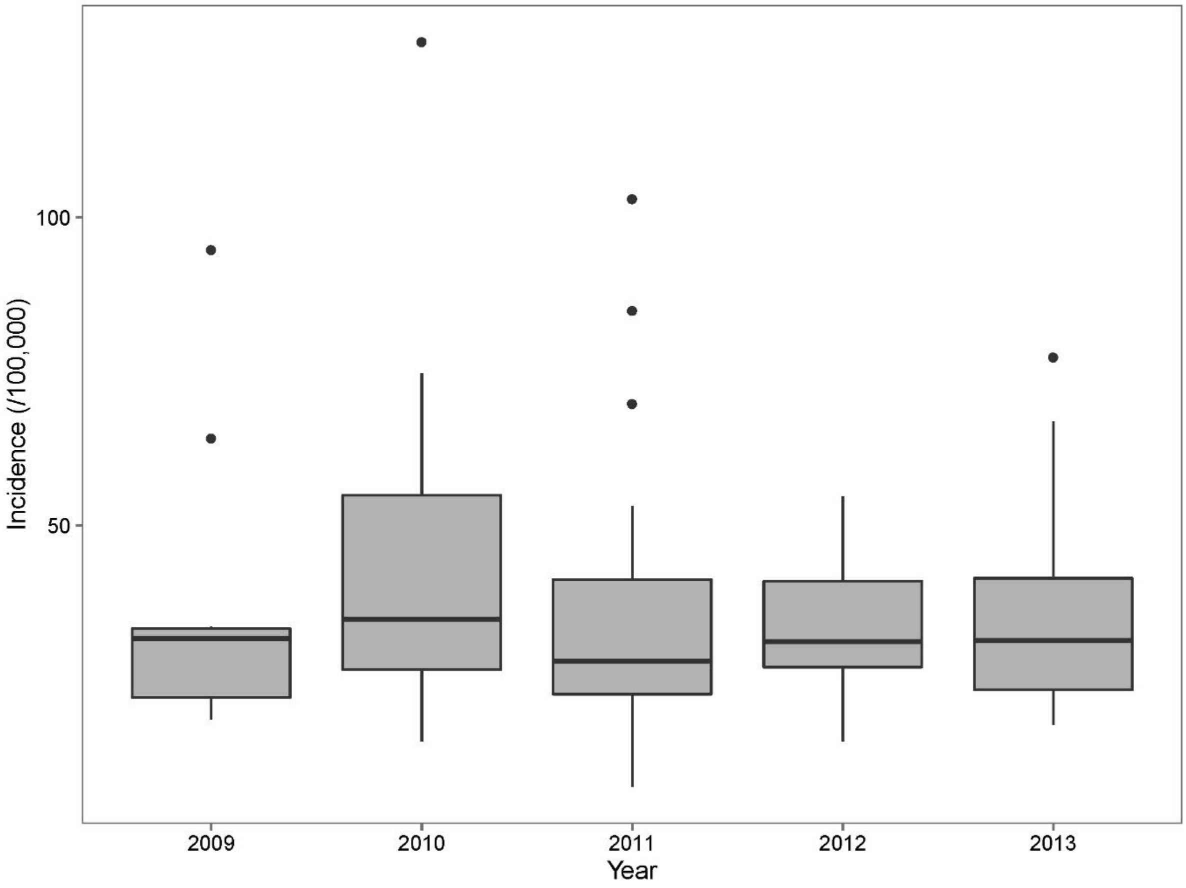

Supplement: Additional file 1: Figure S1. — Study flow chart. Figure S2. Number of culture-confirmed melioidosis cases presenting to Angkor Hospital for Children, 2009–2013. Figure S3. Commune-level geographic distribution of culture-confirmed melioidosis cases from all provinces presenting to Angkor Hospital for Children, 2009–2013. Figure S4. Siem Reap province commune-level geographic distribution of culture-confirmed melioidosis cases admitted to Angkor Hospital for Children, 2009–2013. Figure S5. Siem Reap province commune-level geographic distribution of all patients admitted to Angkor Hospital for Children, 2009–2013. Figure S6. Age distribution of 173 melioidosis cases presenting to Angkor Hospital for Children, 2009–2013. Figure S7. Annual Siem Reap commune level melioidosis incidence estimates in children aged <15 years (PDF 737 kb) [file 12879_2016_2034_MOESM1_ESM.pdf]
